# Municipal distribution of breast cancer mortality among women in Spain

**DOI:** 10.1186/1471-2407-7-78

**Published:** 2007-05-08

**Authors:** Marina Pollán, Rebeca Ramis, Nuria Aragonés, Beatriz Pérez-Gómez, Diana Gómez, Virginia Lope, Javier García-Pérez, Jose Miguel Carrasco, Maria José García-Mendizábal, Gonzalo López-Abente

**Affiliations:** 1 Environmental and Cancer Epidemiology Unit, National Centre for Epidemiology, Carlos III Institute of Public Health, Madrid, Spain; 2 CIBER Epidemiología y Salud Pública (CIBERESP), Spain

## Abstract

**Background:**

Spain has one of the lowest rates of breast cancer in Europe, though estimated incidence has risen substantially in recent decades. Some years ago, the Spanish Cancer Mortality Atlas showed Spain as having a heterogeneous distribution of breast cancer mortality at a provincial level. This paper describes the municipal distribution of breast cancer mortality in Spain and its relationship with socio-economic indicators.

**Methods:**

Breast cancer mortality was modelled using the Besag-York-Molliè autoregressive spatial model, including socio-economic level, rurality and percentage of population over 64 years of age as surrogates of reproductive and lifestyle risk factors. Municipal relative risks (RRs) were independently estimated for women aged under 50 years and for those aged 50 years and over. Maps were plotted depicting smoothed RR estimates and the distribution of the posterior probability of RR>1.

**Results:**

In women aged 50 years and over, mortality increased with socio-economic level, and was lower in rural areas and municipalities with higher proportion of old persons. Among women aged under 50 years, rurality was the only statistically significant explanatory variable.

For women older than 49 years, the highest relative risks were mainly registered for municipalities located in the Canary Islands, Balearic Islands, the Mediterranean coast of Catalonia and Valencia, plus others around the Ebro River. In premenopausal women, the pattern was similar but tended to be more homogeneous. In mainland Spain, a group of municipalities with high RRs were located in Andalusia, near the left bank of the Guadalquivir River.

**Conclusion:**

As previously observed in other contexts, mortality rates are positively related with socio-economic status and negatively associated with rurality and the presence of a higher proportion of people over age 64 years. Taken together, these variables represent the influence of lifestyle factors which have determined the increase in breast cancer frequency over recent decades. The results for the younger group of women suggest an attenuation of the socio-economic gradient in breast cancer mortality in Spain. The geographical variation essentially suggests the influence of other environmental variables, yet the descriptive nature of this study does not allow for the main determinants to be established.

## Background

Breast cancer is the leading malignant tumour in women, accounting for 27% of cancers in European women [1]. Spain has one of the lowest rates in Europe, in terms both of incidence (estimated age-standardised rate of 51 per 100,000 population) more especially, of mortality (16 per 100,000)[1]. The major risk factors seem to be genetic susceptibility, reproductive behaviour, obesity and, less consistently demonstrated, diet[2]. Ecological studies have shown an association with fertility, body weight or fat consumption, but these variables explained only a minor component of the overall variation[3]. A positive association between breast cancer and socio-economic level has been consistently reported [4-10]. Although socio-economic status can be a surrogate of several risk factors (reproductive behaviour, diet or physical activity) [11-13], these variables do not completely explain the excess risk observed in the more affluent groups[8].

One of the classic approaches in epidemiology is the study of geographical distribution. In administrative terms, Spain is divided into Autonomous Regions known as Comunidades Autónomas. These are in turn divided into provinces and, at the lowest level, into municipalities. Breast cancer mortality has been previously studied at a provincial level. The highest standardised rates observed in Las Palmas Province (Canary Islands) were double those registered for Orense, the province with the lowest rate[14].

Currently, spatial epidemiology allows for a greater level of disaggregation. One of the advantages of this approach is to highlight local effects that might be linked to specific geographic, social or environmental characteristics[15]. This study reports on municipal distribution of breast cancer mortality in Spain and the variability associated with socio-economic level and other explanatory variables. Furthermore, given that pre- and postmenopausal tumours have somewhat different risk factors (i.e. obesity seems to act as protective exposure in younger women and is a well-established risk factor among postmenopausal women), the geographical pattern is independently explored in women aged under 50 years or 50 years and over.

## Methods

As our case source, we used all Spanish individual death entries for the period 1989–1998 corresponding to breast cancer (International Classification of Diseases, 9th Revision (ICD-9) code 174) broken down by municipality. A municipality is an administrative unit, made up of a clearly demarcated territory and its population, governed by a municipal council. This is the smallest aggregated division for which mortality and population data could be obtained at a national basis for the study period. Mortality data were furnished by the National Statistics Institute. Municipal populations, broken down by age group (18 groups) and sex, were obtained from the 1991 census and 1996 municipal rolls. These years correspond to the midpoints of the two quinquennia that comprise the study period (1989–1993 and 1994–1998). The person-years for each five-year period were obtained by multiplying these populations by 5.

As an indicator of socio-economic level, the index provided by the Spanish Credit Bank (Banco Español de Crédito) for 1991 was used[16]. This index classifies municipalities into 10 levels, according to different markers of economic activity, namely, the number of holiday homes, bank branch offices and telephones, and estimated average family income. An indicator of rurality was drawn up, based on the number of inhabitants, as classified by the National Statistics Institute in the following 10 categories: > 500,000; 100,001–500,000; 50,001–100,000; 20,001–50,000; 10,001–20,000; 5,001–10,000; 2,001–5,000; 1,001–2,000; 501–1,000; and 101–500 and <100 inhabitants. Finally, the percentage of people over the age of 64 years living in each municipality was deemed to be a surrogate of life-style factors that might have changed and were less prevalent in older generations.

Standardised mortality ratios (SMRs) were computed as the ratio of observed to expected deaths. Expected cases were computed, taking Spanish breast cancer mortality rates, broken down by age and five-year period, as reference. SMRs were also calculated by province and category of explanatory variable, and confidence intervals for these categories were duly computed using Byar's approach[17].

Breast cancer mortality was separately studied among women aged under 50 years and comprised deaths from cancers diagnosed mainly in premenopausal women and among women aged 50 years and over. This latter group was made up of a mixture of pre- and postmenopausal cases but was nevertheless dominated by the second group.

Smoothed municipal relative risks (RRs) were calculated using the conditional autoregressive model introduced by Clayton and Kaldor[18], and further developed by Besag, York and Molliè [19]. This model has been applied in the field of ecological studies[20]. It is a Poisson spatial model with observed cases as the dependent variable, expected cases as offset, and two random effects terms that take the following into account: a) municipal contiguity (spatial term); and b) municipal heterogeneity. Socio-economic level, rurality and percentage of people over the age of 64 years were introduced into the model as continuous explanatory variables. The purpose was twofold: 1) to ascertain their influence on breast cancer mortality; and 2) to smooth relative risk, taking into account the variability associated with these factors rather than merely the spatial relationship among municipal areas. The model took the following form

Oi~Po(Eiλi)log⁡(λi)=α+∑jβj∗xji+hi+bi
 MathType@MTEF@5@5@+=feaafiart1ev1aaatCvAUfKttLearuWrP9MDH5MBPbIqV92AaeXatLxBI9gBaebbnrfifHhDYfgasaacH8akY=wiFfYdH8Gipec8Eeeu0xXdbba9frFj0=OqFfea0dXdd9vqai=hGuQ8kuc9pgc9s8qqaq=dirpe0xb9q8qiLsFr0=vr0=vr0dc8meaabaqaciaacaGaaeqabaqabeGadaaakeaafaqabeGabaaabaGaem4ta80aaSbaaSqaaiabdMgaPbqabaGccqGG+bGFcqWGqbaudaWgaaWcbaGaem4Ba8gabeaakiabcIcaOiabdweafnaaBaaaleaacqWGPbqAaeqaaGGacOGae83UdW2aaSbaaSqaaiabdMgaPbqabaGccqGGPaqkaeaacyGGSbaBcqGGVbWBcqGGNbWzcqGGOaakcqWF7oaBdaWgaaWcbaGaemyAaKgabeaakiabcMcaPiabg2da9iab=f7aHjabgUcaRmaaqababaGae8NSdi2aaSbaaSqaaiabdQgaQbqabaGccqGHxiIkcqWG4baEdaWgaaWcbaGaemOAaOMaemyAaKgabeaakiabgUcaRiabdIgaOnaaBaaaleaacqWGPbqAaeqaaOGaey4kaSIaemOyai2aaSbaaSqaaiabdMgaPbqabaaabaGaemOAaOgabeqdcqGHris5aaaaaaa@5B2B@

where: *λ*_i _is the relative risk in area I; O_i _is the number of deaths in area I; E_i _are the expected cases; *β*_j _is the coefficient representing the effect (log(RR)) of the explanatory variable J, x_ji _refers to the value of the explanatory variable J in the area I, h_i _is the municipal heterogeneity term; and b_i _is the spatial term.

Models were fitted using Bayesian Markov Chain Monte Carlo simulation methods[21]. Posterior distributions of RR were obtained using WinBugs[22]. The criterion of contiguity used was adjacency. Convergence was verified using the BOA (Bayesian Output Analysis) R programme library[23]. Given the great number of parameters, the convergence analysis was performed on a randomly selected sample of 10 towns and cities, taking 4 strata defined by municipal size. Convergence of the estimators was achieved before 100,000 iterations. Risk estimators were computed after a "burn-in" (iterations discarded to ensure convergence) of 300,000 iterations, based on the posterior distribution observed in the next 5,000 iterations.

For comparison purposes, standard Poisson regression models were also fitted, with the three explanatory variables being included to quantify their effect on mortality. Confidence intervals were computed using robust estimates of variance[24], as a way of taking overdispersion into account.

A Geographic Information System was used to plot municipal maps that depicted smoothed RR and the distribution of the posterior probability (pp) that RR>1 (Bayesian equivalent to p value). Insofar as this indicator was concerned, we followed Richardson's criterion[15], which recommends that probabilities above 0.8 should be deemed significant.

## Results

From 1989 to 1998, a total of 56711 breast cancer deaths were registered. Eighty-four percent (47789) corresponded to women aged over 49 years, and the remaining 8922 to younger women. In 3328 municipalities there were no deaths due to this cause. Ninety percent of these villages had less than 1000 inhabitants and the number of expected cases in all of these was lower than 1. Furthermore, 3% of the Spanish population lived in municipalities that registered no breast cancer deaths during the study period. Table 1 displays a number of descriptive statistics, including the distribution of population and explanatory variables, as well as observed and expected cases of breast cancer in the two age groups considered.

**Table 1:** Summaries of population, other explanatory variables and breast cancer mortality in 8077 Spanish towns. Spain, 1989–1998.

	Total	Mean	Standard Deviation	Median	Min	Max	P5 – P95	No. (%) with zero counts
Population	39648759	4908.9	42430.4	586	5	2866850	63 – 14177	0 (0)
Socioeconomic index	-	5.23	2.17	5	1	10	1 – 9	0 (0)
% Population >=65	-	23.4	9.58	22.3	0	100	10.4 – 40.8	0 (0)
Women >=50								
Observed breast cancer cases	47789	5.9	65.8	1	0	4283	0 – 16	3522 (43.6)
Expected number	47789	5.9	60.9	1.0	0.003	4274.5	0.3 – 16.1	0 (0)
SMR	1.00	0.84	2.27	0.53	0	5.85	0.00 – 2.79	3522 (43.6)
Women <50								
Observed breast cancer cases	8922	1.1	11.2	0	0	718	0 – 4	6237 (77.2)
Expected number	8922	1.1	10.5	0.1	0	704.3	0.0 – 3.2	36 (0.5)
SMR	1.00	0.98	6.17	0.0	0	333.3	0.00 – 3.52	6237 (77.2)

**Figure 1:**
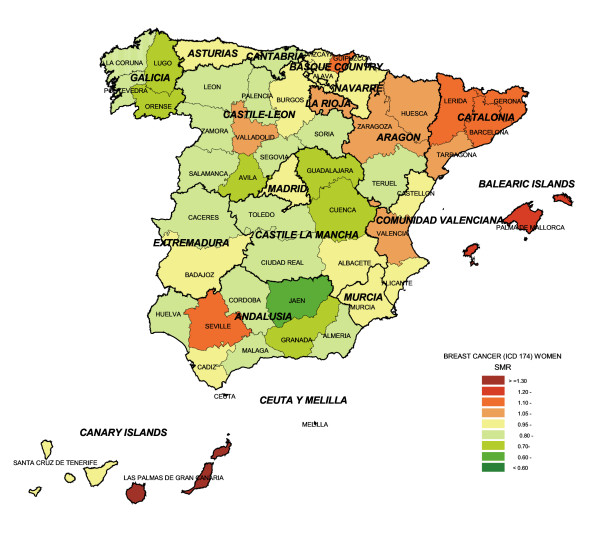
Provincial distribution of breast cancer mortality in women: Spain, 1989–1998.

To give an overall picture, Figure 1 shows breast cancer mortality by province. There were only two provinces with SMRs greater than 1.20, namely, Las Palmas in the Canary Islands (SMR = 1.42), and the Balearic Islands (SMR = 1.21). Jaén in Andalusia registered the lowest mortality (SMR = 0.67). SMRs ranging from 0.70 to 0.80 were observed in Cuenca and Guadalajara (Castile-La Mancha), Lugo and Orense (Galicia), Granada (Andalusia) and Avila (Castile-León).

**Figure 2:**
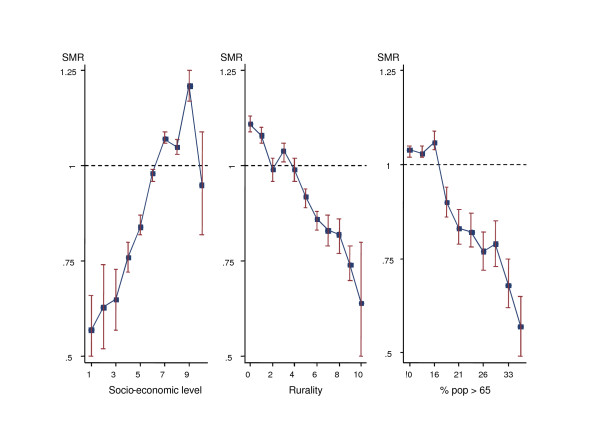
Relationship between breast cancer mortality in women aged 50 years and over (SMRs), and the following three explanatory variables: socio-economic index; rurality; and percentage of subjects over 64 years of age.

Breast cancer mortality in women 50 and more years old:

Figure 2 depicts the relationship between breast cancer mortality in these women and the three explanatory variables. Breast cancer mortality increased with socio-economic level, though a sharp drop is observed in the highest income category. For the other two variables, rurality and percentage of subjects aged over 64 years, a downward trend was observed. Table 2 lists the RR for these explanatory variables, using conventional Poisson regression (left) and the Bayesian model (right). Results in both models were quite similar. Given the trends observed in Figure 2, natural splines were used to investigate possible non-linear trends associated with these variables, but the goodness-of-fit failed to improve.

**Table 2:** Effect of explanatory variables on breast cancer mortality in women over 49 years of age.

Variable	Conventional Poisson regression model		Bayesian model (Besag-York-Molliè)	
	RR	95% Confidence Interval	RR	95% Credibility Interval

Socio-economic index				
per 1 unit	1.069	1.053–1.086	1.046	1.033–1.059
Rurality				
Change per category^1^	0.988	0.975–1.000	0.979	0.972–0.986
% Population >=65				
Change per 10%	0.988	0.984–0.993	0.992	0.992–0.995

1 Categories: 0 = > 500.000 inhabitants, 1 = 100.001–500.000, 2 = 50.001–100.000, 3 = 20.001–50.000, 4 = 10.001–20.000, 5 = 5.001–10.000, 6 = 2.001–5.000, 7 = 1.001–2.000, 8 = 501–1.000, 9 = 101–500, 10 = <100 inhabitants.

**Figure 3:**
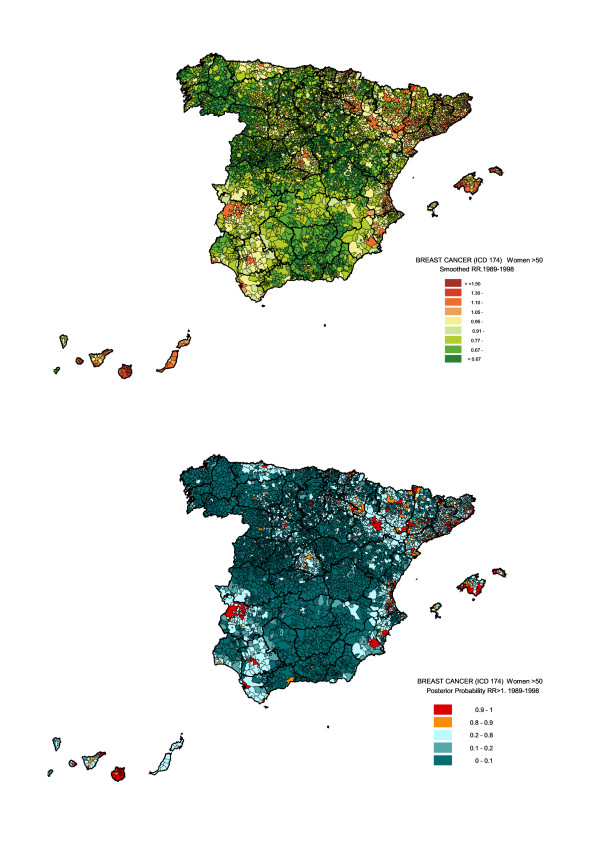
Municipal distribution of breast cancer mortality in women aged 50 years and over: Spain, 1980–1999. Distribution pattern of smoothed relative risk (RR) under the BYM model (top) and posterior probability of RR being greater than 1 (bottom).

Figure 3 shows the smoothed RR map for this age-group (top), together with the distribution of posterior probabilities of having a relative risk greater than 1 (bottom). The highest mortality was registered for the Canary Islands and, to a lesser extent, for the Balearic Islands. On the Spanish mainland, increased mortality was observed along the east coast, chiefly in the Autonomous Regions of Catalonia and Valencia. In Catalonia, RRs were significantly higher than 1 for the four provincial capitals, in the area around Barcelona and on the coast from Tarragona to Torroella de Montgrí. Most municipalities around the Ebro River, downstream from Calahorra onwards, as well as around the Ebro's tributaries, the Cinca and the Segre, are highlighted on the map, and many have posterior probabilities greater than 0.8. In the Autonomous Region of Valencia, excess risks were concentrated along the coast, particularly in Valencia Province. Almost all provincial capitals in northern and eastern Spain registered excess risk, with posterior probabilities greater than 0.9. Finally, some municipalities in Madrid, Extremadura and Andalusia also presented statistically significant, high RRs. Municipalities with RRs greater than 1.25, based on a difference between observed and expected numbers equal to or greater than 3 cases and with a posterior probability equal to or greater than 0.9 are shown in Table 3. Relative risks of over 1.50 were only seen in the Canary Islands. Explanatory variables did not fully account for the excess risk observed in all these municipalities, since their RR were substantially higher than the value obtained by only taking the linear predictor into account, i.e., the part of the model that includes the three explanatory variables.

**Table 3:** Breast cancer mortality in women over 49 years of age in Spain. Towns (grouped by province) having a difference between the number of observed and expected cases equal to or greater than 3, an RR of over 1.25 and a posterior probability (pp) of over 0.9. Spain,1989–1998.

CCAA										
	Province	Municipality	RR	pp	SMR	obs	exp	Rurality	SE	%Pop >65
ANDALUSIA										
	Cadiz	PUERTO DE SANTA MARIA	1.27	0.994	1.49	76	50.94	2	6	7.74
	Sevilla	SAN JUAN DE AZNALFARACHE	1.31	0.992	1.84	39	21.24	3	6	10.17
ARAGON										
	Zaragoza	UTEBO	1.26	0.907	1.77	12	6.78	5	7	9.37
BALEARIC ISLANDS										
	Mallorca	CALVIA	1.47	0.998	1.42	22	15.50	3	9	7.02
	Mallorca	PALMA DE MALLORCA	1.41	1.000	1.43	502	350.25	1	8	13.27
	Mallorca	ANDRAITX	1.32	0.938	1.32	13	9.88	5	9	15.91
	Menorca	CIUTADELLA DE MENORCA	1.32	0.941	1.39	30	21.56	3	6	12.16
	Ibiza	EIVISSA	1.28	0.961	1.45	37	25.44	3	8	9.09
	Mallorca	ESPORLES	1.28	0.960	2.06	9	4.37	6	8	18.18
	Mallorca	FELANITX	1.27	0.983	1.52	31	20.44	4	8	19.03
CATALONIA										
	Barcelona	VILAFRANCA DEL PENEDES	1.33	0.996	1.43	49	34.35	3	8	13.79
		GIRONELLA	1.32	0.945	1.95	17	8.73	5	7	22.00
		VILANOVA I LA GELTRU	1.32	0.997	1.40	79	56.39	3	7	13.76
		CABRILS	1.30	0.976	2.06	7	3.41	6	9	10.49
		BARBERA DEL VALLES	1.29	0.987	1.64	33	20.09	3	7	6.93
		CABRERA DE MAR	1.28	0.961	2.08	6	2.88	6	9	10.03
		VILASSAR DE MAR	1.27	0.975	1.45	20	13.83	4	8	11.93
		S. MARGARIDA I ELS MONJOS	1.26	0.974	1.79	7	3.90	6	8	11.69
		SANT PERE DE RIBES	1.25	0.961	1.30	18	13.82	4	7	10.14
	Gerona	BEGUR	1.46	0.986	2.25	7	3.11	6	9	11.52
		TORROELLA DE MONTGRI	1.40	0.993	1.63	14	8.58	5	9	14.27
		PALAMOS	1.38	0.992	1.48	25	16.91	5	9	13.01
		PALAFRUGELL	1.33	0.987	1.23	26	21.16	4	8	14.46
		PUIGCERDA	1.32	0.942	1.39	11	7.90	5	8	14.63
		CALONGE	1.31	0.983	1.78	12	6.73	5	9	14.67
	Lerida	SOLSONA	1.31	0.930	1.53	13	8.52	5	8	16.24
		TARREGA	1.26	0.987	1.72	27	15.74	4	7	18.24
	Tarragona	CALAFELL	1.38	0.989	1.63	15	9.22	4	8	14.1
COMUNIDAD VALENCIANA										
	Alicante	SANTA POLA	1.36	0.962	1.59	25	15.71	4	6	10.79
	Valencia	BENIPARRELL	1.34	0.959	4.30	6	1.40	7	10	11.77
CANARY ISLANDS										
	Las Palmas	ARUCAS	1.63	1.000	1.90	49	25.75	3	5	9.97
		TELDE	1.63	1.000	1.70	91	53.40	2	5	6.82
		PALMAS DE GRAN CANARIA	1.58	1.000	1.56	516	330.42	1	6	9.4
		SANTA BRIGIDA	1.58	1.000	1.83	22	11.99	4	6	9.58
		INGENIO	1.52	0.999	1.50	24	16.01	3	5	6.81
		SAN BARTOLOME DE TIRAJANA	1.49	1.000	1.42	23	16.16	3	6	5.57
		FIRGAS	1.47	0.999	1.71	10	5.84	5	5	10.53
		TEROR	1.43	0.999	1.32	15	11.33	4	5	11.68
		VALSEQUILLO DE GRAN CANARIA	1.43	0.999	2.04	11	5.40	5	5	9.79
		MOYA	1.41	0.998	1.96	17	8.68	5	5	12.25
		SANTA LUCIA	1.39	0.992	1.24	25	20.15	3	5	5.83
		GALDAR	1.37	0.992	1.39	26	18.64	3	5	9.47
	Tenerife	SAN SEBASTIAN DE LA GOMERA	1.52	0.939	2.27	12	5.28	5	6	10.17
		ARONA	1.38	0.985	1.67	25	14.93	3	7	6.12
BASQUE COUNTRY										
	Guipuzcoa	HONDARRIBIA	1.26	0.957	1.68	25	14.85	4	7	11.53

CCAA = Autonomous Community. RR = estimated relative risk. SMR = standard mortality ratio. pp = posterior probability that RR>1. SE = socio-economic index.

**Figure 4:**
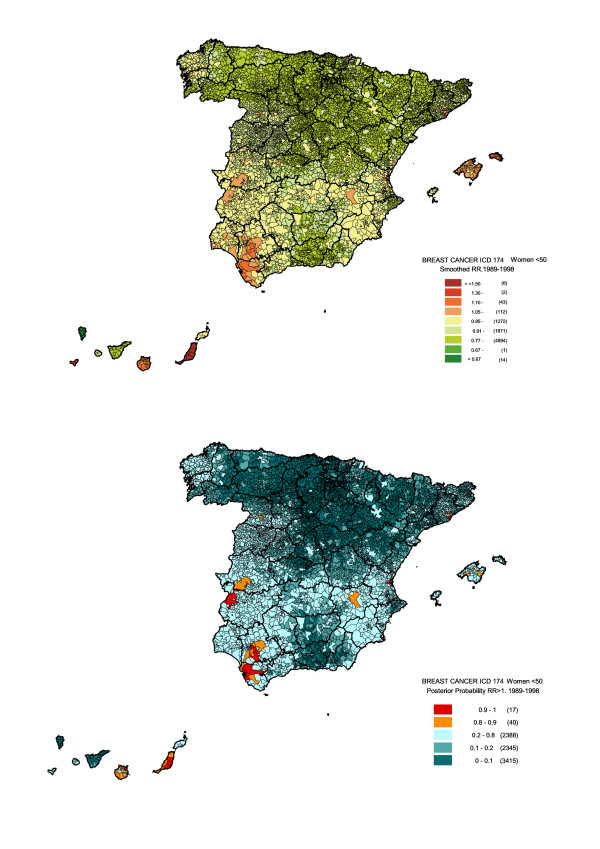
Municipal distribution of breast cancer mortality in women under 50 years of age: Spain, 1980–1999. Distribution pattern of smoothed relative risk (RR) under the BYM model (top) and posterior probability of RR being greater than 1 (bottom).

Breast cancer mortality in women younger than 50 years:

Observed deaths among premenopausal women accounted for only 16% of overall breast cancer mortality, and 6237 municipalities registered zero cases (Table 1). While no clear trend was associated with socio-economic level or percentage of population above the age of 64, SMRs were inversely associated with rurality (data not shown). This information is summarised by RR estimators of these factors (Table 4). As rurality was the only statistically significant variable, the final model only included this explanatory term. The geographical pattern (Figure 4) was less marked than in older women, with a somewhat greater risk in Canary Islands, Balearic Islands, the city of Valencia and south-west Andalusia (Cadiz and Seville). In this last-mentioned area, there was a cluster of municipalities with higher risk, located near the left bank of the Guadalquivir River, including towns and cities such as Jerez de la Frontera, El Puerto de Santa María, Puerto Real and Chiclana in Cadiz, and Utrera, Seville, Alcalá de Guadaira and Dos Hermanas in Seville. Table 5 shows those municipalities with RRs equal to or greater than 1.10, a posterior probability of over 0.80 and a difference between observed and expected cases equal to or greater than 3.

**Table 4:** Effect of the explanatory variables on breast cancer mortality in women younger than 50.

Variable	Conventional Poisson regression model		Bayesian model (Besag-York-Molliè)	
**Three factor model**	RR	95% Confidence Interval	RR	95% Credibility Interval

Socio-economic index				
per 1 unit	1.002	0.974–1.029	0.992	0.967–1.016
Rurality				
Change per category^1^	0.978	0.965–0.992	0.978	0.965–0.991
% Population >=65				
Change per 10%	1.004	0.998–1.011	1.004	0.998–1.011
**Final model**				
Rurality				
Change per category^1^	0.981	0.968–0.993	0.982	0.977–0.993

1 Categories: 0 = > 500.000 inhabitants, 1 = 100.001–500.000, 2 = 50.001–100.000, 3 = 20.001–50.000, 4 = 10.001–20.000, 5 = 5.001–10.000, 6 = 2.001–5.000, 7 = 1.001–2.000, 8 = 501–1.000, 9 = 101–500, 10 = <100 inhabitants.

**Table 5:** Breast cancer mortality in women under 50 years of age in Spain. Towns (grouped by province) having a difference between the number of observed and expected cases equal to or greater than 3, an RR greater than 1.10 and a posterior probability (pp) of over 0.8. Spain, 1989–1998.

CCAA								
	Province	Municipality	RR	pp	SMR	Obs	Exp	Rurality
ANDALUSIA								
	Cadiz	CHICLANA DE LA FRONTERA	1.16	0.928	1.90	19	9.99	3
		JEREZ DE LA FRONTERA	1.13	0.967	1.27	52	40.78	1
		EL PUERTO DE SANTA MARIA	1.14	0.912	1.33	21	15.76	2
		PUERTO REAL	1.13	0.907	1.60	11	6.86	3
		ROTA	1.11	0.805	1.63	8	4.91	3
		SAN FERNANDO	1.16	0.925	1.37	26	19.03	2
	Sevilla	ALCALA DE GUADAIRA	1.12	0.932	1.40	17	12.12	2
		LOS PALACIOS Y VILLAFRANCA	1.11	0.848	1.50	9	6.01	3
		SEVILLA	1.15	0.995	1.16	186	160.87	0
		UTRERA	1.11	0.919	1.90	17	8.94	3
BALEARIC ISLANDS								
	Mallorca	MANACOR	1.13	0.803	1.50	9	6.02	3
CATALONIA								
	Barcelona	BARCELONA	1.18	1.000	1.25	487	389.01	0
		TERRASSA	1.10	0.920	1.30	49	37.62	1
CANARY ISLANDS								
	Las Palmas	LAS PALMAS DE GRAN CANARIA	1.15	0.942	1.10	91	82.99	1
		TELDE	1.15	0.895	1.21	21	17.43	2
		TUINEJE	1.62	0.919	3.63	5	1.38	5
COMUNIDAD VALENCIANA								
	Valencia	VALENCIA	1.14	0.994	1.19	219	183.79	0

CCAA = Autonomous Community. RR = estimated relative risk. SMR = standard mortality ratio. pp = posterior probability that RR>1.

## Discussion

Our results reflect excess breast cancer mortality in women aged over 49 years in the Canary Islands, Balearic Islands, Catalonia and Valencia. Furthermore, high significant excess risks were observed in municipalities of La Rioja, Navarre, Aragon and Catalonia around the Ebro River. Most provincial capitals in the north and east of Spain displayed increased RR. Lastly, Badajoz, Mérida and Almendralejo in Extremadura, Seville and Alcalá de Guadaira in the Province of Seville, and Puerto Real and Puerto de Santa María in Cadiz registered high, significant RRs. The geographical pattern in younger women appeared to be more uniform and no RR over 1.20 were in evidence. The only exception was Tuineje (Las Palmas). On the Spanish mainland, a group of municipalities with high RRs were located in two Andalusian provinces, Cadiz and Seville, near the Guadalquivir River.

When seeking possible etiological clues, it could be argued that incidence would be more valuable than mortality, given that in Spain breast cancer survival rates are quite high, i.e., around 78% at five years[25]. Nevertheless, mortality is the only global source of information available in this country. Breast cancer is a well-certified cause of death in Spain, with both detection and confirmation rates exceeding 90%[26]. Incidence rates would not be useful for comparison purposes during the study period, since Spanish Autonomous Regions put their screening programmes in place during the 1990s. Screening artificially increases incidence, by advancing diagnosis of very small tumours and including cases that will not progress[27]. In view of regional differences in the starting point of screening programmes around Spain, extracting conclusions based on incidence would be problematic in this particular period. Mortality rates, on the other hand, are not influenced by such overdiagnosis. Although it would be interesting to study the influence of screening programmes on breast cancer mortality, the time-frame renders this impossible because, in almost all cases, the first round of screening was only completed after 1995 in the territories in question.

Municipal RRs were estimated, including three ecological variables in the model, namely: the socio-economic index provided by the Spanish Credit Bank; the number of inhabitants categorised by the National Statistics Institute; and the percentage of subjects aged over 64 years. These variables were used as markers of the heterogeneous distribution of lifestyle and reproductive factors influencing breast cancer frequency. Selection of these variables was limited by the availability of information at a municipal level, so they only partially reflect the distribution of breast cancer risk factors in Spain. However, their inclusion served to smooth relative risks, taking into account both the spatial relation among municipalities and the variability associated with these indices. In the model proposed by Besag, York and Molliè, modelling the clustering variation allows for unmeasured risk factors that vary smoothly with location. Where the pattern of covariate variation is similar to that of disease risk, location may act as a confounder[20]. This phenomenon will produce a change in the regression coefficient on introduction of the spatial clustering term. In our study, this location-induced confounding effect is small, as can be seen by comparing the estimated effects yielded by Besag, York and Molliè models against those yielded by conventional Poisson regression (see Tables 2 and 4).

Small area analysis tends to reduce ecological fallacy, since the populations defined by municipal boundaries are more homogeneous. This might well be true of villages and towns of average size. In large cities, however, the results reported here correspond to an overall mean, and socio-economic and mortality differences inside cities have been disregarded. It could be very interesting to assess whether such differences exist in major Spanish cities, such as Madrid, Barcelona, Valencia, Seville and Zaragoza. This internal variability could not been explored in this paper.

Risk factors for breast cancer may differ for premenopausal and postmenopausal cancers [2]. Family history and hereditary susceptibility are particularly relevant for premenopausal tumours [28,29], whereas obesity and reproductive behaviour are generally more important in postmenopausal women. The geographical pattern of premenopausal breast cancer was designated as a specific goal of the study. One third of breast cancer diagnoses in Spain occur in premenopausal women [30] and breast cancer mortality before age 50 may be considered as due to premenopausal cancers [31]. The geographical distribution observed for the younger group, albeit attenuated, was no different to that observed for older women. The most distinctive result was the higher risk registered in south-west Andalusia, near the course of the Guadalquivir River.

Research indicates that use of well-chosen census tract socio-economic measures can provide important insights into socio-economic disparities in the burden of breast cancer[32]. In our study, using the economic classification provided by the Spanish Credit Bank, a positive gradient of mortality was observed. Although socio-economic class is a poorly understood concept that has not been homogeneously measured[7], a positive association between this factor and breast cancer has consistently been reported [4-7,9,10]. Socio-economic status can be related to environmental exposures and reproductive behaviours (e.g., nulliparity)[7,11]. In terms of mortality, however, greater survival, coupled with higher participation in screening programmes[32], have been observed in more affluent groups[7]. In Europe, the ELDCARE study has shown a strong correlation between breast cancer survival and national gross domestic product (correlation coefficient of 0.80) [33]. Spanish survival rates are greater than those expected from this correlation, probably reflecting lower inequalities in access to adequate medical services[33]. Yet women living in most affluent municipalities might receive earlier diagnosis and treatment, and this would explain the comparatively lower SMR observed for the highest socio-economic category (see Figure 2).

Socio-economic level was not associated with breast cancer mortality in younger women. This result is in agreement with the few studies available that looked into changes in social inequalities in breast cancer mortality[31,34,35]. Social disparities are decreasing more quickly in younger groups[31]. One explanation could be better prognosis in young women from affluent areas, due to their greater awareness and access to medical diagnosis, since these age groups are not included in mass screening programmes. Alternatively, the lack of socio-economic differences in younger women may reflect a more uniform pattern vis-à-vis lifestyle factors. Thus, parity has decreased in France more markedly among women with lower educational levels[31]. Likewise, in Finland the decline in educational differences in breast cancer mortality has been associated with changes in reproductive behaviour and body mass index among less educated women[35].

In our study, breast cancer mortality in both age groups was higher in more urban areas, which is consistent with the increased incidence observed in urban settings[8]. Lower incidence in rural areas probably reflects differences in education, age at first birth and other breast-cancer-related factors. On the other hand, when considering mortality, real differences in incidence may be attenuated, since lower participation rates in screening programmes have been reported in rural areas[32] and this translates as a lower number of in situ tumours and a higher proportion of more advanced stages with increased lethality[36]. Rurality was the only explaining factor negatively associated with mortality in women under the age of 50 years, probably indicating that changes in lifestyle risk factors occurred earlier in urban areas.

A negative correlation was observed between the percentage of old population and breast cancer mortality in women aged 50 years or over. Birth-cohort trends reflect demographic patterns that are related to recognised breast cancer risk factors[37]. Age-cohort-period analysis of breast cancer mortality across time has consistently shown a strong variation associated with the cohort component, in Europe [38] and in Spain[39]. An analysis performed among carriers of BRCA1 and BRCA2 mutations, showed a greater incidence of breast cancer in younger birth-cohorts compared with relatives of these women belonging to older generations[40]. This indicates that, even among mutation carriers, lifestyle factors more prevalent in younger cohorts determine the overall risk. Lifestyle changes have been remarkable in Spain. During the first half of the 1990s, Spain had the lowest birth rate in Europe[41]. Furthermore, the decline in fertility proved particularly sharp in Spain, where the mean birth rate dropped from 2.86 in 1970 to 1.18 in 1995[41]. The reasons for this rapid decrease were: improved quality of life and education; increased contraceptive use; and the sheer speed of social change nationwide[41]. There has also been a massive influx of women into the workforce and, due to high unemployment, children now tend to live longer in the parental home than in the past[41]. In terms of dietary patterns, there is evidence to show that rapid urbanisation and the growing proportion of females in the active workforce have led to important changes in food patterns in recent decades, with an increase in consumption of animal products, such as meat, fish, milk and dairy products, fats and processed foods. In contrast, there has been a decrease in consumption of cereals, potatoes and legumes[42,43]. These changes can be assumed to be linked to younger generations. Given the length of the study period, an age-cohort-period analysis was not advisable. The percentage of population aged over 64 years was chosen to reflect the presence of older generations, which could be deemed both an indicator of the relative weight of elder cohorts in these municipalities, and a marker of the social and cultural environment, on the assumption that such an environment would modulate the speed of change. It is possible, however, that part of the observed association between breast cancer mortality and the proportion of people aged over 64 years living in any given area may be somewhat spurious, resulting from the comparison of SMRs across regions with different age structures. If this were the case, the inclusion of this variable in the model would enable smoothed RRs to be obtained, taking this distribution into account.

Although the three explanatory variables are interrelated, all were retained in the final model for older women, showing that, to a certain extent, they act as surrogates for different risk factors. Nevertheless, RRs for municipalities with high breast cancer mortality seemed to be higher than the predicted trend that had been estimated according to these variables. This implies the intervention of other conditions not properly captured by these ecological variables. Postmenopausal women with high body mass index have an increased risk[2]. Fourteen percent of Spanish women are obese and a further 28% are overweight[44]. Obesity is more prevalent in Andalusia, Extremadura, Castile-La Mancha and the Canary Islands[44]. Yet, obesity would not seem to explain the observed geographical pattern. Insofar as nutrition is concerned, over the past 100 years, the mean age of menarche has declined from 16 to 14 years in all industrialised nations[45,46]. This phenomenon has been predominantly attributed to abundant nutrition during childhood[47]. In this regard, the higher risk in the Balearic and Canary Islands can be partly linked to the higher proportion of European immigrants who come from countries with higher incidence of breast cancer and now live in these regions. These women have spent their childhood in areas with higher background rates.

At an individual level, well-established breast-cancer risk factors account for less than 50% of overall incidence[48]. Like most chronic diseases, breast cancer is considered to be the result of an interaction between genetic and environmental factors[29,49]. Hereditary susceptibility plays an important role in breast cancer pathogenesis, but time-trends as well as twin-studies suggest that in sporadic breast cancer, environmental, nutritional and lifestyle factors dominate over genetic predisposition[47,49]. Environmental factors are believed to account for a large proportion of cases. Apart from ionising radiation, a well-established risk factor, some chemicals induce mammary cancer in rodents, though evidence in humans is lacking[50]. For instance, to show that in rats, cadmium at very low doses acts as an oestrogen mimic, indicating a need to investigate the effects of metals on breast cancer risk[51]. Pesticides are also of interest because many mimic oestrogen or cause mammary tumours in animals[52,53]. The most abundant of these contaminants are the pesticide, dichloro-diphenyl-trichloroethane (DDT), and polychlorinated biphenyls (PCBs)[50]. They are included among persistent organic compounds, given that they degrade slowly, bioaccumulate and may be found in human adipose tissue, blood and breast milk. The most prevalent organic compound residues found in human tissues are dichlorodiphenyldichloroethane (DDE) -the major metabolite of DDT- and PCBs. Available epidemiological evidence linking pesticides and breast cancer is considered inadequate[50]. A recent meta-analysis concluded that, taken together, available results provide enough evidence to discard a relationship between breast cancer and DDT[54], and organochlorine exposure is not believed to be causally related to breast cancer[55]. Nevertheless, these studies provide no information on exposure during critical periods of human development, namely, from conception to adolescence[54]. In Spain, even though DDT was banned in 1977, a recent study showed that 99% of the 682 samples collected from healthy people in the Canary Islands displayed detectable levels of some DDT-derivatives[56]. The presence of a very high DDT/DDE ratio indicated chronic exposure to DDT, which has persisted until now[56]. The highest levels were seen in Gran Canaria, the island on which the municipalities with the highest RR are located. However, given the current state of knowledge, any implication of DDT and DDE levels in the excess mortality observed in Gran Canaria is highly speculative.

High breast cancer mortality was also observed along the Ebro river in postmenopausal women and near the left bank of the Guadalquivir River in premenopausal women. Both rivers are included among fluvial areas with chemical pollution in Spain[57]. The Ebro river has received a great amount of pollutants, including pesticides, hydrocarbons and nitrates[57]. A recent study shows that organochlorine contaminants continue to be of concern in this area and that, among its degradation products, DDT still predominates, indicating recent inputs of this banned substance[58].

## Conclusion

Even though breast cancer mortality has begun to decline in Spain, a heterogeneous geographical pattern is in evidence, with higher rates in the Canary Islands, the Balearic Islands, the Mediterranean coast of Catalonia and Valencia, and a series of municipalities around the Ebro River. Among premenopausal women, increased mortality was also observed for municipalities located along the last third of the Guadalquivir River. As has been previously reported in other contexts, mortality rates are positively associated with socio-economic status and negatively associated with rurality and the presence of a higher proportion of persons over the age of 64 years (born before 1931). Taken together, these variables represent the influence of lifestyle factors that have determined the increase in breast cancer frequency over recent decades. On the other hand, the results for the younger group of women would suggest an attenuation of the socio-economic gradient in breast cancer mortality. The geographical variation mainly suggests the influence of other environmental variables, though a heterogeneous distribution of genetic factors cannot be ruled out. The descriptive nature of this study means, however, that the main determinants of this pattern cannot be ascertained.

## Abbreviations

DDE: dichlorodiphenyldichloroethane; DDT: dichlorodiphenyltrichloroethane; ICD-9: international classification of diseases, 9th revision; PCBs: polychlorinated biphenyls; RR: relative risk; SMR: standardised mortality ratio.

## Competing interests

The author(s) declare that they have no competing interests.

## Authors' contributions

MP, GLA, NA, and BPG were all involved in designing the study. GLA, RR & MP performed the statistical analysis. DG draw the maps using GIS software. VL & JGP reviewed the information about possible pollutant sources in the areas with high RR, JMC & MJG reviewed the socio-economic index used in this study and the abundant literature on this issue. MP wrote the draft of the manuscript to which all authors subsequently contributed. All authors made contribution to statistical analyses and interpretation of results, and revised the manuscript for important intellectual content. All authors read and approved the final manuscript.

## Pre-publication history

The pre-publication history for this paper can be accessed here:



## References

[B1] Ferlay J, Bray F, Pisani P, Parkin DM (2004). GLOBOCAN 2002: Cancer Incidence, Mortality and Prevalence Worldwide [IARC CancerBase No 5 version 20,].

[B2] Dumitrescu RG, Cotarla I (2005). Understanding breast cancer risk – where do we stand in 2005?. J Cell Mol Med.

[B3] Parkin DM (2004). International variation. Oncogene.

[B4] Faggiano F, Partanen T, Kogevinas M, Boffetta P (1997). Socioeconomic differences in cancer incidence and mortality.

[B5] Heck KE, Wagener DK, Schatzkin A, Devesa SS, Breen N (1997). Socioeconomic status and breast cancer mortality, 1989 through 1993: An analysis of education data from death certificates. American Journal of Public Health.

[B6] Dano H, Andersen O, Ewertz M, Petersen JH, Lynge E (2003). Socioeconomic status and breast cancer in Denmark. International Journal of Epidemiology.

[B7] Baquet CR, Commiskey P (2000). Socioeconomic factors and breast carcinoma in multicultural women. Cancer.

[B8] Robert SA, Strombom I, Trentham-Dietz A, Hampton JM, McElroy JA, Newcomb PA (2004). Socioeconomic risk factors for breast cancer – Distinguishing individual- and community-level effects. Epidemiology.

[B9] Pollan M, Gustavsson P (1999). High-risk occupations for breast cancer in the Swedish female working population. American Journal of Public Health.

[B10] Sarfati D, Blakely T, Shaw C, Cormack D, Atkinson J (2006). Patterns of disparity: ethnic and socio-economic trends in breast cancer mortality in New Zealand. Cancer Causes Control.

[B11] dos Santos I, Beral V (1997). Socioeconomic differences in reproductive behaviour.

[B12] Heck KE, Pamuk ER (1997). Explaining the relation between education and postmenopausal breast cancer. American Journal of Epidemiology.

[B13] Dunnell K, Bunting J, Wood R, Babb P (1999). Measuring aspects of women's life and work for the study of variations in health. American Journal of Industrial Medicine.

[B14] Lopez-Abente G, Pollan M, Escolar A, Errezola M, Abraira V (2001). Atlas of cancer mortality and other causes of death in Spain, 1978–1992.

[B15] Richardson S, Thomson A, Best N, Elliott P (2004). Interpreting posterior relative risk estimates in disease-mapping studies. Environmental Health Perspectives.

[B16] Banco Español de Credito (1993). Anuario del Mercado Español.

[B17] Breslow NE, Day NE (1987). Statistical methods in cancer research The design and analysis of cohort studies.

[B18] Clayton D, Kaldor J (1987). Empirical Bayes Estimates of Age-Standardized Relative Risks for Use in Disease Mapping. Biometrics.

[B19] Besag J, York J, Mollie A (1991). Bayesian Image-Restoration, with 2 Applications in Spatial Statistics. Annals of the Institute of Statistical Mathematics.

[B20] Clayton DG, Bernardinelli L, Montomoli C (1993). Spatial Correlation in Ecological Analysis. International Journal of Epidemiology.

[B21] Gilks W, Richardson S, Spiegelhalter D (1995). Markov Chain Montecarlo in practise Interdisciplinary statistics.

[B22] Spiegelhalter D, Thomas A, Best N (2003). WinBUGS user manual Versioin 141.

[B23] Smith BJ (2001). Bayesian Output Analysis Program (BOA), Version 0991 for S-Plus and R.

[B24] Hardin J, Hilbe J (2001). Generalized Linear Models and extensions.

[B25] Sant M, Aareleid T, Berrino F, Bielska Lasota M, Carli PM, Faivre J (2003). EUROCARE-3: survival of cancer patients diagnosed 1990–94 – results and commentary. Annals of Oncology.

[B26] Perez-Gomez B, Aragones N, Pollan M, Suarez B, Lope V, Llacer A (2006). Accuracy of cancer death certificates in Spain: A summary of available information. Gaceta Sanitaria.

[B27] Zahl PH, Strand H, Maehlen J (2004). Incidence of breast cancer in Norway and Sweden during introduction of nationwide screening: prospective cohort study. British Medical Journal.

[B28] Anderson DE (1992). Familial Versus Sporadic Breast-Cancer. Cancer.

[B29] Risch N (2001). The genetic epidemiology of cancer: Interpreting family and twin studies and their implications for molecular genetic approaches. Cancer Epidemiology Biomarkers & Prevention.

[B30] Martin M, Llombart-Cussac A, Lluch A, Alba E, Munarriz B, Tusquets I (2004). Epidemiological study of the GEICAM group about breast cancer in Spain (1990–1993): El Alamo project. Medicina Clinica.

[B31] Menvielle G, Leclerc A, Chastang JF, Luce D (2006). Social inequalities in breast cancer mortality among French women: disappearing educational disparities from 1968 to 1996. Br J Cancer.

[B32] Bigby J, Holmes MD (2005). Disparities across the breast cancer continuum. Cancer Causes Control.

[B33] Quaglia A, Vercelli M, Lillini R, Mugno E, Coebergh JW, Quinn M (2005). Socio-economic factors and health care system characteristics related to cancer survival in the elderly. A population-based analysis in 16 European countries (ELDCARE project). Crit Rev Oncol Hematol.

[B34] Wagener DK, Schatzkin A (1994). Temporal Trends in the Socioeconomic Gradient for Breast-Cancer Mortality Among Us Women. American Journal of Public Health.

[B35] Martikainen P, Valkonen T (2000). Diminishing educational differences in breast cancer mortality among Finnish women: a register-based 25-year follow-up. Am J Public Health.

[B36] Schootman M, Fuortes LJ (1999). Breast and cervical carcinoma: the correlation of activity limitations and rurality with screening, disease incidence, and mortality. Cancer.

[B37] Lacey JV, Devesa SS, Brinton LA (2002). Recent trends in breast cancer incidence and mortality. Environ Mol Mutagen.

[B38] La Vecchia C, Negri E, Levi F, Decarli A (1997). Age, cohort-of-birth, and period-of-death trends in breast cancer mortality in Europe. J Natl Cancer Inst.

[B39] Lopez-Abente G, Pollan M, Aragones N, Perez-Gomez B, Llacer A, Perez J (2002). Tendencias de la mortalidad en España, 1952–1996 Efecto de la edad, de la cohorte de nacimiento y del periodo de muerte.

[B40] King MC, Marks JH, Mandell JB (2003). Breast and ovarian cancer risks due to inherited mutations in BRCA1 and BRCA2. Science.

[B41] Bosch X (1998). Investigating the reasons for Spain's falling birth rate. Lancet.

[B42] Serra-Majem L, Ribas L, Lloveras G, Salleras L (1993). Changing patterns of fat consumption in Spain. Eur J Clin Nutr.

[B43] Aranceta J (2001). Spanish food patterns. Public Health Nutr.

[B44] Ministerio de Sanidad (2003). Encuesta Nacional de Salud 2003.

[B45] Merzenich H, Boeing H, Wahrendorf J (1993). Dietary-Fat and Sports Activity As Determinants for Age at Menarche. American Journal of Epidemiology.

[B46] Stoll BA (1998). Western diet, early puberty, and breast cancer risk. Breast Cancer Research and Treatment.

[B47] Gerber B, Muller H, Reimer T, Krause A, Friese K (2003). Nutrition and lifestyle factors on the risk of developing breast cancer. Breast Cancer Res Treat.

[B48] Madigan MP, Ziegler RG, Benichou J, Byrne C, Hoover RN (1995). Proportion of Breast-Cancer Cases in the United-States Explained by Well-Established Risk-Factors. Journal of the National Cancer Institute.

[B49] Lichtenstein P, Holm NV, Verkasalo PK, Iliadou A, Kaprio J, Koskenvuo M (2000). Environmental and heritable factors in the causation of cancer – Analyses of cohorts of twins from Sweden, Denmark, and Finland. New England Journal of Medicine.

[B50] Coyle YM (2004). The effect of environment on breast cancer risk. Breast Cancer Res Treat.

[B51] Johnson MD, Kenney N, Stoica A, Hilakivi-Clarke L, Singh B, Chepko G (2003). Cadmium mimics the in vivo effects of estrogen in the uterus and mammary gland. Nature Medicine.

[B52] Soto AM, Sonnenschein C, Chung KL, Fernandez MF, Olea N, Serrano FO (1995). The E-Screen Assay As A Tool to Identify Estrogens – An Update on Estrogenic Environmental-Pollutants. Environmental Health Perspectives.

[B53] Gammon DW, Aldous CN, Carr WC, Sanborn JR, Pfeifer KF (2005). A risk assessment of atrazine use in California: human health and ecological aspects. Pest Management Science.

[B54] Lopez-Cervantes M, Torres-Sanchez L, Tobias A, Lopez-Carrillo L (2004). Dichlorodiphenyldichloroethane burden and breast cancer risk: A meta-analysis of the epidemiologic evidence. Environmental Health Perspectives.

[B55] Calle EE, Frumkin H, Henley SJ, Savitz DA, Thun MJ (2002). Organochlorines and breast cancer risk. Ca-A Cancer Journal for Clinicians.

[B56] Zumbado M, Goethals M, Alvarez-Leon EE, Luzardo OP, Cabrera F, Serra-Majem L (2005). Inadvertent exposure to organochlorine pesticides DDT and derivatives in people from the Canary Islands (Spain). Science of the Total Environment.

[B57] Barea J, de Armas H, Caballero MJ, Carrasco JF, Colmenarejo P, Garcia L La calidad de las aguas en España Un estudio por cuencas.

[B58] Gomez-Gutierrez AI, Jover E, Bodineau L, Albaiges J, Bayona JM (2006). Organic contaminant loads into the Western Mediterranean Sea: Estimate of Ebro River inputs. Chemosphere.

